# Structural and catalytic properties of the peroxygenase P450 enzyme CYP152K6 from *Bacillus methanolicus*

**DOI:** 10.1016/j.jinorgbio.2018.08.002

**Published:** 2018-11

**Authors:** Hazel M. Girvan, Harshwardhan Poddar, Kirsty J. McLean, David R. Nelson, Katherine A. Hollywood, Colin W. Levy, David Leys, Andrew W. Munro

**Affiliations:** a Centre for Synthetic Biology of Fine and Specialty Chemicals (SYNBIOCHEM), Manchester Institute of Biotechnology, School of Chemistry, The University of Manchester, Manchester M1 7DN, United Kingdom; b Department of Microbiology, Immunology and Biochemistry, University of Tennessee Health Science Center, Memphis, TN 38163, United States of America

**Keywords:** Peroxygenase, Cytochrome P450, Organic products, Protein structure, Substrate binding, EPR spectroscopy

## Abstract

The CYP152 family of cytochrome P450 enzymes (P450s or CYPs) are bacterial peroxygenases that use hydrogen peroxide to drive hydroxylation and decarboxylation of fatty acid substrates. We have expressed and purified a novel CYP152 family member – CYP152K6 from the methylotroph *Bacillus methanolicus* MGA3. CYP152K6 was characterized using spectroscopic, analytical and structural methods. CYP152K6, like its peroxygenase counterpart P450_SPα_ (CYP152B1) from *Sphingomonas paucimobilis*, does not undergo significant fatty acid-induced perturbation to the heme spectrum, with the exception of a minor Soret shift observed on binding dodecanoic acid. However, CYP152K6 purified from an *E. coli* expression system was crystallized and its structure was determined to 1.3 Å with tetradecanoic acid bound. No lipids were present in conditions used for crystallogenesis, and thus CYP152K6 must form a complex by incorporating the fatty acid from *E. coli* cells. Turnover studies with dodecanoic acid revealed several products, with 2-hydroxydodecanoic acid as the major product and much smaller quantities of 3-hydroxydodecanoic acid. Secondary turnover products were undec-1-en-1-ol, 2-hydroxydodec-2-enoic acid and 2,3-dihydroxydodecanoic acid. This is the first report of a 2,3-hydroxylated fatty acid product made by a peroxygenase P450, with the dihydroxylated product formed by CYP152K6-catalyzed 3-hydroxylation of 2-hydroxydodecanoic acid, but not by 2-hydroxylation of 3-hydroxydodecanoic acid.

## Highlights

• The cytochrome P450 (P450 or CYP) CYP152K6 from *Bacillus methanolicus* was characterized.
• CYP152K6 is a peroxygenase that catalyzes efficient oxidation of dodecanoic acid.
• A 1.3 Å crystal structure was solved for CYP152K6 bound to tetradecanoic acid.
• EPR reveals three low-spin CYP152K6 species whose populations change on lipid binding.
• CYP152K6 produces 2,3-dihydroxydodecanoic acid as a novel peroxygenase product.

## Graphical abstract

*Bacillus methanolicus* CYP152K6 is a novel cytochrome P450 (P450 or CYP) peroxygenase family member. Its tetradecanoic acid-bound crystal structure reveals that the substrate binds through its carboxylate to a conserved arginine residue. Hydrogen peroxide displaces the distal water on the heme, forming a reactive iron-oxo species that catalyzes substrate oxidation.

**Figure:**
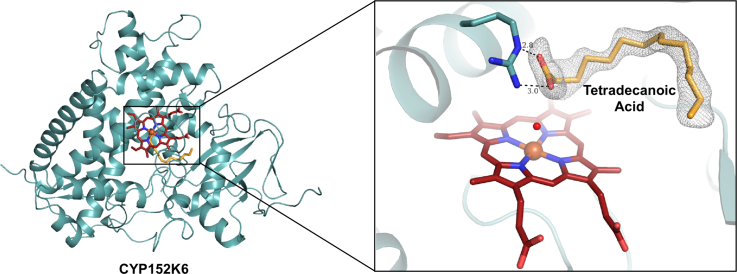

## 1 Introduction

The cytochromes P450 (P450s or CYPs) are a superfamily of monooxygenase enzymes found in organisms from archaea and bacteria through to man. They catalyze a vast array of reactions that, in most cases, result from the formation of a highly reactive heme iron-oxo species known as compound I [1]. The compound I is a ferryl (Fe^IV^=O) porphyrin radical cation that can efficiently insert its oxygen atom into a substrate molecule bound close to the heme in the active site of the enzyme [2]. According to the specific P450 enzyme, the particular substrate bound and its binding site, the reactions catalyzed can include hydroxylation, epoxidation, desaturation, demethylation and other dealkylation reactions, reduction, decarboxylation and sulfoxidation [3]. Most P450s require electron transfer from one or more redox partner proteins in order to generate compound I. In bacterial systems these are typically soluble, NAD(P)H-dependent ferredoxin reductases and their cognate ferredoxins, although flavodoxins were also shown to support selected bacterial P450s [[4], [5], [6]]. In eukaryotes, the enzyme cytochrome P450 reductase (CPR or POR), a natural fusion protein combining NADPH-ferredoxin reductase-like and flavodoxin-like domains, provides electrons for the function of the P450s [7]. The CPR and P450s are both bound to cellular membranes through N-terminal transmembrane alpha-helices. P450s are also found in the mitochondria of eukaryotic cells, including those in the adrenal glands. In these cases, the P450s are reduced by the NADPH-dependent, FAD-binding adrenodoxin reductase and the 2Fe—2S cluster-binding adrenodoxin protein [8,9].

While most P450 enzymes function catalytically by using NAD(P)H-dependent redox partners, a particular class of bacterial P450 enzymes that can function efficiently using hydrogen peroxide (H_2_O_2_) was identified approximately two decades ago [10,11]. These “peroxygenases” from the CYP152 P450 family short circuit the canonical P450 catalytic cycle by reacting hydrogen peroxide (H_2_O_2_) with the ferric, substrate-bound form of the P450, and by converting this species directly to the ferric-hydroperoxo state (also known as compound 0). This process obviates the necessity for the delivery of two electrons (from a redox partner, which would form consecutively the ferric-superoxo and ferric-peroxo intermediates) and of a proton (that would convert the ferric-peroxo intermediate into compound 0). In the peroxygenase P450 cycle, a further proton delivery to compound 0 results in its rapid dehydration and its conversion to compound I [12,13]. This process is referred to as the “peroxide shunt” and has been used widely in studies of P450 enzymes [14]. However, it is often an inefficient method for driving P450 catalysis and one in which oxidative modification of the heme and protein side chains occurs alongside any productive reactions in which H_2_O_2_ reacts with the heme iron to form compound 0. In contrast, the peroxygenase P450s have been shown to be efficient catalysts that convert fatty acids of various chain lengths to hydroxylated species and, for some of the peroxygenases, to terminal alkenes through an oxidative decarboxylation reaction [15,16]. The first peroxygenase P450s to be expressed and characterized were P450_SPα_ (CYP152B1) from the aerobic, Gram-negative Bacillus *Sphingomonas paucimobilis*, and P450_BSβ_ (CYP152A1) from the Gram-positive *Bacillus subtilis*. P450_SPα_ was initially shown to catalyze exclusively the hydroxylation of fatty acids at the alpha-position, whereas P450_BSβ_ hydroxylated fatty acids at both the alpha- and beta-positions, with beta-hydroxylated fatty acids being the major product [11,17]. Subsequently, other such peroxygenases have been expressed and characterized. These include a P450 from *Clostridium acetobutylicum* (CYP152A2), which favours hydroxylation of fatty acids at the alpha-position [18]. More recently, Rude et al. revisited the properties of P450_BSβ_ and investigated other peroxygenases from *Jeotgalicoccus* sp. 8456, *Corynebacterium efficiens*, *Kocuria rhizophila* and *Methylobacterium populi*, and found that each of these peroxygenases could produce the terminal alkene 1-pentadecene from palmitic (hexadecanoic) acid substrate (as well as alpha- and/or beta-hydroxylated hexadecanoic acid in most cases) [16]. The production of terminal alkenes is of potential importance with respect to biofuel manufacture.

In this manuscript, we report the expression, purification, spectroscopic and structural analysis, and product characterization for CYP152K6 from the methylotroph *Bacillus methanolicus*. CYP152K6 is a previously uncharacterized member of the CYP152 P450 peroxygenase family which is able to hydroxylate at both the alpha and beta carbons on its fatty acid substrate. CYP152K6 co-purifies with tetradecanoic acid bound in the active site, enabling the analysis of the unusual binding mode of this lipid.

## 2 Materials and methods

### 2.1 Materials

Isopropyl-β-d-thiogalactopyranoside (IPTG) was obtained from Formedium (Hunstanton, UK). LB bacterial growth medium and kanamycin sulfate were from Thermo Fisher Scientific (Warrington, UK). Fatty acids and derivatives (dodecanoic acid, 2-hydroxydodecanoic acid, 3-hydroxydodecanoic acid, tetradecanoic acid, 12-methyltetradecanoic acid, hexadecanoic acid, octadecanoic acid, 1-undecene, 3-indole propionic acid, 4-phenylbutyric acid, 1,2,3,4-tetrahydro-2-naphthoic acid, arachidonic acid, phytanic acid, hexanoic acid, octanoic acid), lysozyme, DNase I, potassium chloride, potassium phosphate, anhydrous magnesium sulfate, *N*,*O*-Bis(trimethylsilyl)trifluoroacetamide (BSTFA), Trimethylchlorosilane (TMCS), SigmaFAST protease inhibitor cocktail tablets (EDTA-free), imidazole, 4-phenylimidazole and hydrogen peroxide were from Merck Chemicals Ltd. (Southampton, UK). Restriction enzymes (*Nde*I and *Bam*HI) were from NEB (Hitchin, UK). Ni-NTA resin was from Generon (Slough, UK). Carbon monoxide and nitrogen gases were from BOC (Guildford UK). Protein crystallization screens were from Molecular Dimensions (Newmarket, UK).

### 2.2 Identification, cloning and expression of CYP152K6

**Fig. 1:**
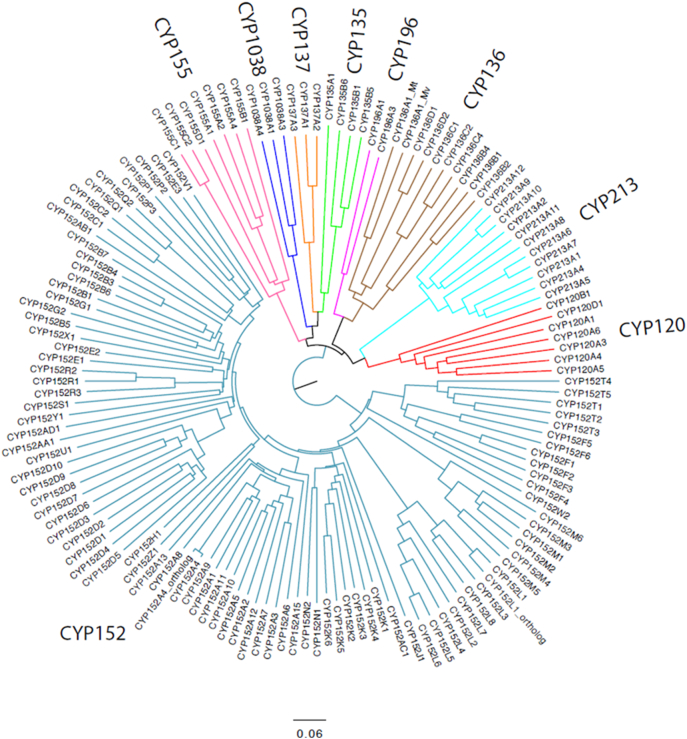
CYP152 family neighbour-joining tree. The tree shows 93 CYP152 family peroxygenase P450 sequences, in addition to 47 other sequences from related P450 families. The tree was computed by the CLUSTAL Omega server at EBI (http://www.ebi.ac.uk/Tools/msa/clustalo/). The tree was midpoint rooted, drawn in Figtree v1.3.1 and labelled in Adobe Illustrator CC.

The *CYP152K6* gene from the methylotroph *Bacillus methanolicus* MGA3 (Uniprot ID I3DZK9; NCBI reference WP_003349199) was identified using a protein BLAST search with the amino acid sequence of the well-studied OleT_JE_ enzyme (CYP152L1) from *Jeotgalicoccus* sp. 8456. The gene title was assigned by Prof. David Nelson according to the level of similarity of the CYP152K6 amino acid sequence with other members of the CYP152 P450 peroxygenase family. A phylogenetic tree of CYP152 family members (as well as those of other related bacterial P450 families) is shown in Fig. 1.

### 2.3 Gene synthesis and cloning of the CYP152K6 gene

The gene encoding the CYP152K6 protein from *Bacillus methanolicus* was codon optimized for overexpression in *E. coli* and synthesised by GeneArt with the addition of an N-terminal 6xHis-tag and a TEV protease cleavage site between the *CYP152K6* gene and its His-tag. The gene construct was sub-cloned into the pET24b expression vector between the *Nde*I and *Bam*HI restriction sites.

### 2.4 Expression and purification of the *B. methanolicus* CYP152K6 enzyme

The CYP152K6-pET24b plasmid DNA construct was transformed into *E. coli* BL21 (DE3) competent cells containing 30 μg/mL kanamycin (for plasmid selection) to enable the overexpression of the CYP152K6 protein. 4 × 500 mL of LB growth medium were inoculated with 5 mL of an overnight culture of the transformant cells and bacterial cell growth was continued at 37 °C until an OD_600_ of 0.8 was reached. Thereafter, 1 mM IPTG was added to induce expression of the *CYP152K6* gene in the *E. coli* cells. The temperature was then lowered to 25 °C and cell growth was continued for a further 18 h prior to harvesting the cells. The *E. coli* cells were harvested by centrifugation at 6000 rpm, 4 °C, using a JLA-8.1000 rotor in a Beckman-Coulter Avanti J-26 XP centrifuge. The cell pellet was resuspended in 100 mL of 100 mM potassium phosphate buffer (pH 8.0) containing 200 mM KCl (buffer A). To this suspension, 2 SigmaFAST protease inhibitor cocktail tablets (EDTA-free) were added together with lysozyme and DNase I (both at 100 μg/mL). The *E. coli* cells were lysed by 20 rounds of sonication at 40% power using a Bandelin Sonopuls sonicator, with each round lasting 10 s followed by a 2 min rest period, and with the sample maintained in an ice-cold environment throughout. The lysate was then centrifuged to remove cell debris using a JA25.50 rotor in the Beckman-Coulter Avanti J-26 XP centrifuge at 18000 rpm for 30 min at 4 °C. The clarified supernatant was mixed with Ni-NTA affinity resin (Generon), using 10 mL resin/100 g of cell pellet pre-equilibrated in buffer A, and the mixture stirred for 3 h at 4 °C. The resin was then packed into a column and washed with 20 column volumes (CV) of buffer A. Non-tagged proteins were removed by washing the column with 20 CV buffer A containing 100 mM imidazole, prior to eluting the CYP152K6 protein in buffer A containing 200 mM imidazole. The 6xHis-Tag was then cleaved by overnight incubation at 4 °C with ~1000 U of S219 V TEV protease expressed from plasmid pRK793 (Addgene plasmid no. 8827). The pRK793 vector expresses the catalytic domain of the tobacco etch virus (TEV) protease as a fusion protein with maltose binding protein. The fusion protein self-cleaves to produce the TEV protease catalytic domain with a N-terminal His-tag and a C-terminal polyarginine tag [19]. The peptide containing the TEV cleavage site and any non-cleaved CYP152K6 were removed by dialysis of the protein into buffer A without imidazole and by reapplying the protein to the Ni-NTA column. CYP152K6 bound weakly to the column and was eluted with buffer A containing 20 mM imidazole. Protein was then concentrated to ~35 mg/mL prior to loading onto a Superdex 200 16/600 column (GE Healthcare, Little Chalfont, UK) pre-equilibrated in buffer A for a final purification step using an AKTA Pure chromatography system (GE Healthcare). Fractions were assessed for purity by UV–visible spectroscopy and by SDS-PAGE. The fractions of highest purity were pooled, concentrated to ~15 mg/mL and then either immediately flash frozen in liquid nitrogen for later use, or used immediately for protein crystallization studies.

### 2.5 Spectroscopic analysis of the CYP152K6 enzyme

UV–visible spectroscopic analysis of CYP152K6 was done using a Cary 60 spectrophotometer (Agilent Technologies Ltd., Cheadle, UK) using a 1 cm quartz pathlength cuvette containing 1 mL of buffer A with the P450 protein (typically in the concentration range of ~2–12 μM) at 25 °C. Anaerobic measurements were made using a Cary 60 spectrophotometer housed in an anaerobic glove box (Belle Technology UK Ltd., Weymouth, UK) under a nitrogen environment with oxygen concentration maintained at less than 1 ppm. Anaerobic buffer was prepared by extensively bubbling buffer A with oxygen-free nitrogen. Carbon monoxide saturated buffer was prepared by extensively bubbling a sealed tube of anaerobic buffer A with carbon monoxide gas. Sodium dithionite was added to CYP152K6 in CO-saturated buffer in order to prepare the ferrous/CO-bound form of the enzyme. Substrate and inhibitor binding titration studies were done using a Cary 60 UV–visible spectrophotometer, and by the sequential addition of 0.1 μL aliquots of substrate (fatty acids) or inhibitor (4-phenylimidazole) compounds prepared as 20 mM stocks with ethanol as the solvent.

Continuous Wave (CW) X-band electron paramagnetic resonance (EPR) spectra were collected at a temperature of 10 K using a Bruker ELEXSYS 500 EPR spectrometer equipped with an ER 4122SHQ Super High Q cavity. Temperature control was effected using an Oxford Instruments ESR900 cryostat connected to an ITC 503 temperature controller. Microwave power was 0.5 mW, modulation frequency was 100 kHz, and modulation amplitude was 5 G. EPR spectra were collected for CYP152K6 at a protein concentration of 220 μM in the as-purified form, and following the addition of dodecanoic (lauric) acid to CYP152K6 at final concentrations of 1 mM and 2 mM.

### 2.6 Analysis of products formed by CYP152K6-dependent oxidation of fatty acid substrates

Fatty acid substrate turnover reactions were carried out in buffer A in a total volume of 500 μL. Reactions contained 1 μM CYP152K6, 200 μM substrate (one of: dodecanoic acid, 2-hydroxydodecanoic acid, 3-hydroxydodecanoic acid, tetradecanoic acid, hexadecanoic acid and 1-undecene from a 20 mM stock in ethanol) and were initiated by the addition of 400 μM H_2_O_2_. Reactions were allowed to proceed for 30 min before stopping the reaction by the addition of 25 μL of 37% HCl and by the extraction of remaining substrate and product in an equal volume of dichloromethane. Residual aqueous material was removed from the dichloromethane by the addition of anhydrous magnesium sulfate. Trimethyl silyl derivatives were formed by the addition of a 1:1 volume of BSTFA (*N*,*O*-Bis[trimethylsilyl]trifluoroacetamide) containing 1% TMCS (trimethylchlorosilane) and by incubation of the mixture at 60 °C for 1 h. Standards of the dodecanoic acid, tetradecanoic acid and hexadecanoic acid substrates, and of the 2-hydroxylated, 3-hydroxylated or decarboxylated (n-1 alkene) products of each fatty acid were prepared at a final concentration of 200 μM in dichloromethane prior to derivatization by the same method as detailed for the turnover reactions. Following derivatization, 1 μL of sample was injected onto an Agilent 59756 GC/MSD with 7890B GC, installed with a VF-5HT 30 m × 250 mm × 0.25 mm column. The front inlet was set at 250 °C, and a split ratio of 10:1 was used with column flow set at 1.2 mL/min. The oven was held at 60 °C for 2 min before being ramped to 325 °C at 15 °C/min, where it was held for 2 min. Electronic ionization was used, and *m*/*z* ratios of 50–550 were recorded at 50 Hz and 230 °C.

### 2.7 Crystallization and structural refinement of *Bacillus methanolicus* CYP152K6

The 6xHis-tag of the purified enzyme was cleaved by incubating the tagged form of CYP152K6 with TEV protease. The protein was then concentrated to ~15 mg/mL in 0.1 M potassium phosphate (pH 8.0) containing 0.2 M KCl. Drops containing a total volume of 400 nL (1:1, enzyme:mother liquor) were set up using various commercially available crystallization screens (Molecular Dimensions). Initial crystals were obtained in a condition from the screen JCSG Plus (condition D5: 0.1 M HEPES pH 7.5, 70% v/v 2-methyl-2,4-pentanediol [MPD]). These crystals were used to prepare seed stocks and another round of screening was performed with seeding in drops of 400 nL volume (150 nL protein, 50 nL seed stock, 200 nL mother liquor). Crystals appeared in a condition from the Morpheus screen (condition A5) and were used for X-ray diffraction studies. The crystals were harvested and frozen directly by plunging them into liquid nitrogen, and were kept frozen until required for data collection.

The enzyme crystallized in the space group *C*2 with one monomer per asymmetric unit. Sequence similarity searches showed that CYP152K6 shares ~50% identity with P450_BSβ_. The structure of P450_BSβ_ (PDB: 1IZO) [20] was used as model for molecular replacement in Phaser-MR [21] from the PHENIX software suite [22]. Iterative cycles of AutoBuild [23] were performed to generate a complete model. Subsequently, cycles of refinement were performed in phenix.refine [24] in conjunction with manual model building in COOT [25]. The final R/R_free_ values are 17.3/19.3%. Analysis of the structure and figure preparation was done using Pymol. The final model of CYP152K6 in complex with tetradecanoic acid has been deposited with the PDB with the code 6FYJ.

## 3 Results

### 3.1 Bioinformatics analysis of CYP152K6

The *Bacillus methanolicus CYP152K6* gene was identified as a member of the CYP152 family and assigned to the CYP152K subfamily of P450 enzymes. Fig. 1 shows a neighbour-joining tree computed by the CLUSTAL Omega server at EBI (http://www.ebi.ac.uk/Tools/msa/clustalo/), with 93 CYP152 sequences and 47 other sequences from related P450 families. The tree was midpoint rooted, drawn in Figtree v1.3.1 and labelled in Adobe Illustrator CC.

### 3.2 Production of *Bacillus methanolicus* CYP152K6 in an *E. coli* expression system

The *Bacillus methanolicus CYP152K6* gene was codon optimized for overexpression in *E. coli* and was found to express efficiently in *E. coli* BL21 (DE3) cells using a pET24b plasmid vector, as described in 2.3, 2.4 above. Following harvest of bacterial cells, cell disruption was achieved effectively by sonication, with proteolysis of CYP152K6 prevented by use of a cocktail of protease inhibitors. CYP152K6 was purified in three steps involving (i) binding of the His-tagged CYP152K6 to Ni-NTA resin and removal of contaminants using buffer A containing 100 mM imidazole, prior to elution of CYP152K6 using 200 mM imidazole; (ii) TEV protease cleavage of the CYP152K6 His-tag and reapplication of the cleaved enzyme to the Ni-NTA column, followed by its elution using buffer A containing 20 mM imidazole; and (iii) a final size exclusion chromatography step using a Superdex 200 16/60 column on an AKTA purifier system to generate highly purified CYP152K6. A typical yield of the CYP152K6 enzyme was approximately 20 mg/L using this method. CYP152K6 is a monomer as established by gel-filtration analysis, and is also observed as a monomer in its crystallized form (see Section 3.5).

### 3.3 UV–visible and EPR spectroscopic analysis of CYP152K6

**Fig. 2:**
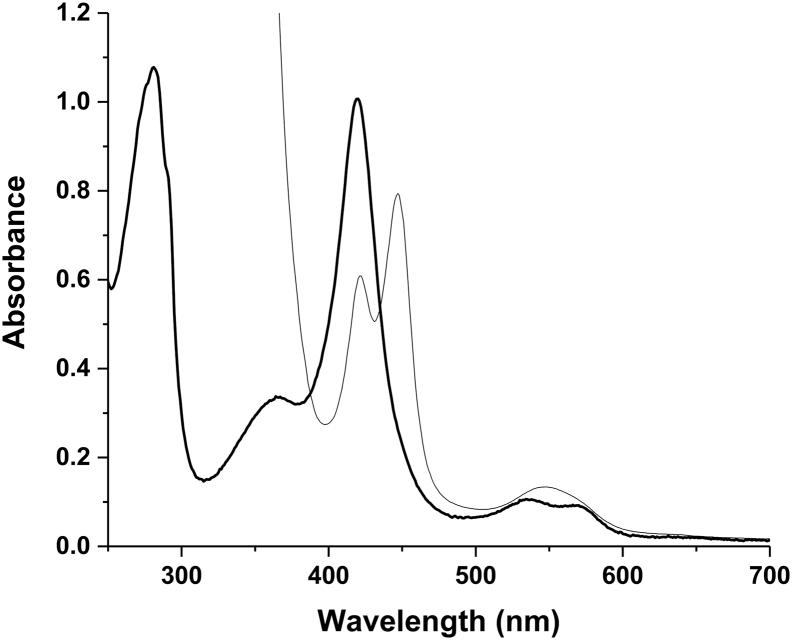
Conversion of CYP152K6 to a ferrous-CO complex. The thick black line is the native CYP152K6 (11 μM). The thin line shows the spectrum collected following CYP152K6 reduction with sodium dithionite in CO-saturated buffer A. Soret spectral maxima are seen at 447 nm (P450 form) and at 420 nm (P420 form) for the Fe^II^—CO complex. A peak at 546 nm is formed in the Q-band region. The P450:P420 ratio is ~80:20%. The extended spectrum for the oxidized form of CYP152K6 includes the A_280_ band, which is of similar absorbance intensity to the Soret band. Using the Protparam tool from the Expasy Bioinformatics Resource Portal (https://www.expasy.org/), an A_280_ extinction coefficient was calculated as 81.4 mM^−1^ cm^−1^. This coefficient is similar to that determined for the CYP152K6 Soret band (ε_418_ = 91 mM^−1^ cm^−1^), indicating a high level of purity of the CYP152A6 enzyme.

Reduction of CYP152K6 with sodium dithionite under anaerobic conditions in the presence of carbon monoxide resulted in the conversion of the spectrum to the Fe^II^—CO form, with Soret features at both 447 nm and 420 nm, and a small band in the visible region at ~546 nm (Fig. 2). This is indicative of two distinct species with different proximal ligands to the CYP152K6 heme iron. The 447 nm species (P450) is ligated by cysteine thiolate, while the 420 nm species (P420) is likely coordinated by cysteine thiol. Studies by Ogura et al. demonstrated that the binding of the substrate epothilone D could convert the P420 state of the *Sorangium cellulosum* P450 EpoK enzyme (CYP167A1) to the P450 form, indicating that a cysteine thiol-to-thiolate conversion occurs in the P420 state of the enzyme in response to substrate binding [26]. Similarly, the binding of the steroid estriol was shown to retard the P450-to-P420 transition that occurs following the formation of the P450 complex of the *Mycobacterium tuberculosis* sterol demethylase CYP51B1 [27]. In other studies on the cholesterol oxidizing CYP142A1 enzyme from *M. tuberculosis*, the binding of the substrate cholest-4-en-3-one induced an almost complete P420-to-P450 conversion of the Fe^II^—CO complex at pH 8.0 [28]. These data indicate that the P420 species involved are not inactive/denatured states of their respective enzymes, but are instead enzyme forms that are in equilibrium with the P450 state and which can be converted towards the P450 state through e.g. substrate addition or by alteration of buffer pH.

**Fig. 3:**
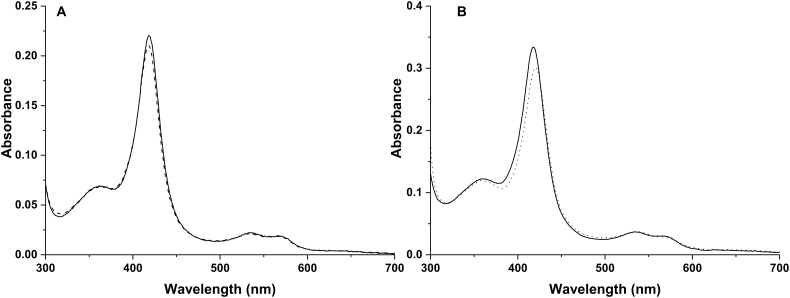
Substrate and inhibitor binding to CYP152K6. Panel A shows purified, substrate-free CYP152K6 (2.3 μM, solid line spectrum) and after binding to 400 μM dodecanoic acid (dashed line spectrum). Dodecanoic acid consistently induces a small shift in the CYP152K6 Soret spectrum. Panel B shows CYP152K6 (3.5 μM, solid line spectrum) and after saturation with the azole compound 4-phenylimidazole (dotted line spectrum). A Soret shift from 418 to 421 nm is observed, with an apparent 4-phenylimidazole *K*_d_ of 110 ± 5 μM.

UV–visible spectroscopy of the ferric (resting) state of CYP152K6 shows a typical P450 spectrum with the Soret band at 418 nm, and the smaller alpha and beta bands at 568 and 535 nm, respectively. These spectral features are consistent with a predominantly low-spin ferric form of the heme iron, with cysteine thiolate and water as the proximal and distal ligands to the heme iron (Fig. 3A). Reduction of CYP152K6 with sodium dithionite results in only a partial conversion to a ferrous state of the enzyme, even after extensive incubation under anaerobic conditions. The binding of a number of lipid substrates (dodecanoic acid, 2-hydroxydodecanoic acid, 3-hydroxydodecanoic acid, tetradecanoic acid, 12-methyltetradecanoic acid, hexadecanoic acid, octadecanoic acid, 1-undecene, 3-indole propionic acid, 4-phenylbutyric acid, 1,2,3,4-tetrahydro-2-naphthoic acid, arachidonic acid, phytanic acid, hexanoic acid, octanoic acid) to the ferric form of CYP152K6 did not elicit any significant perturbation to its spin-state equilibrium, as judged by the apparent absence of change in its UV–visible spectrum. A similar phenomenon was observed for the *Sphingomonas paucimobilis* P450_SPα_ enzyme on binding to fatty acids [29]. However, a small Soret absorbance shift was observed on the binding of dodecanoic acid (from 418 to 417.5 nm) (Fig. 3A). The binding of the heme-coordinating inhibitor 4-phenylimidazole resulted in a decrease in intensity of the heme Soret band and a red shift of the peak from 418 to 421 nm at apparent ligand saturation. Azole inhibitors often induce more substantial Soret band shifts (e.g. to ~424–425 nm), although in this case the extent of the Soret shift is likely affected by the presence of tetradecanoic acid bound in the CYP152K6 active site (see Section 3.5) (Fig. 3B).

**Fig. 4:**
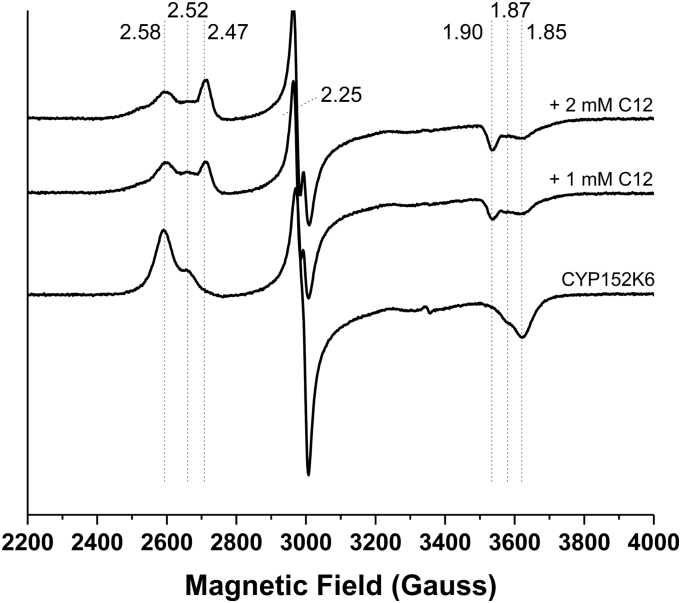
EPR spectroscopic analysis of CYP152K6. X-band EPR spectra are shown for native CYP152K6 (220 μM) and for the enzyme bound to 1 mM and 2 mM dodecanoic acid (C12). Three different sets of low-spin EPR signals are seen in each case at *g* = 2.58/2.25/1.85; 2.52/2.25/1.87; and 2.47/2.25/1.90. Addition of the fatty acid substrate causes shifts in the equilibria between the three different species, while the positions of the *g*-values themselves remain essentially the same after substrate binding.

X-band EPR spectroscopy of the substrate-free resting form of CYP152K6 revealed a rhombic EPR spectrum that is typical of a low-spin, ferric P450. Three low-spin species are evident for the as-purified form of CYP152K6 with *g*-values (*g*_z_/*g*_y_/*g*_x_) = 2.58/2.25/1.85; 2.52/2.25/1.87; 2.47/2.25/1.90. The first of these species (*g* = 2.58/2.25/1.85) is predominant for the substrate-free form of CYP152K6. However, on the addition of dodecanoic acid (at 1 mM and 2 mM final concentrations) the relative proportions of the low-spin components change, with the *g* = 2.47/2.25/1.90 species becoming the dominant form at 2 mM dodecanoic acid (Fig. 4). Thus, the addition of dodecanoic acid alters the equilibria between the three major components in the low-spin EPR spectrum, but does not change their *g*-values significantly. There is negligible signal associated with a high-spin heme iron component in any of these spectra. The EPR spectroscopic properties of CYP152K6 are similar to those of P450_SPα_, with the substrate-free form having *g*-values of 2.59/2.25/1.85. On addition of myristic acid (tetradecanoic acid, C14:0), a second low-spin species emerges (of similar magnitude to the first species) with *g*-values of 2.53/2.25/1.84 [29]. These data appear confirmatory of a similar electronic environment in the peroxygenases CYP152K6 and P450_SPα_, which is distinct from those of typical bacterial P450 monooxygenases (e.g. 2.42/2.26/1.92 for the heme domain of *Bacillus megaterium* flavocytochrome P450 BM3 [CYP102A1], and 2.41/2.24/1.92 for the *Bacillus subtilis* P450 BioI [CYP107H1]) [30,31].

In recent studies we have characterized the *Jeotgalicoccus* sp. 8456 peroxygenase OleT_JE_, which produces mainly terminal alkene products from fatty acid substrates [16,32,33]. OleT_JE_ requires high salt conditions (0.75 M NaCl) to remain in a soluble state. However, this is not the case for CYP152K6, which is stable in solution at much lower salt concentrations.

### 3.4 Analysis of products formed by CYP152K6-dependent oxidation of fatty acids

**Fig. 5:**
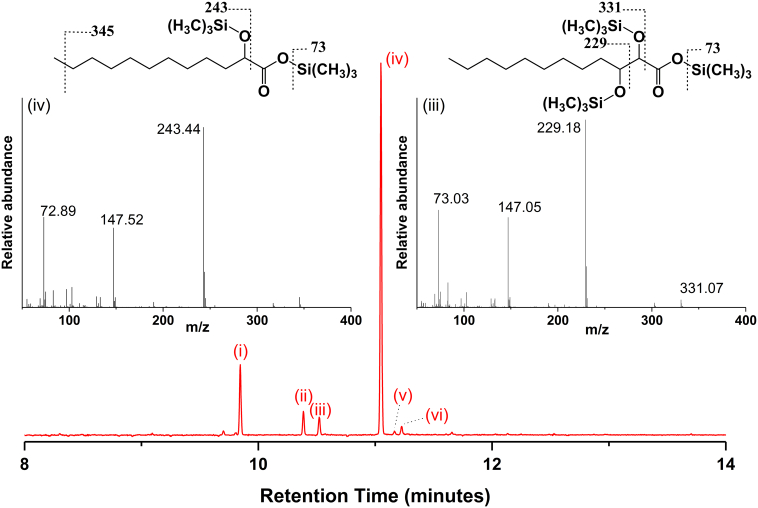
GC–MS of dodecanoic acid turnover by CYP152K6. The main figure shows the gas chromatogram of dodecanoic acid turnover in red. The peaks relate to the trimethyl silyl derivatives of: (i) dodecanoic acid, (ii) 2,3-hydroxydodecanoic acid, (iii) undec-1-en-1-ol, (iv) 2-hydroxydodecanoic acid, (v) 3-hydroxydodecanoic acid, and (vi) 2-hydroxydodec-2-enoic acid. Shown as insets are the ion spectra corresponding to peaks (iv) – left and (v) – right. Mass/charge ratios are indicated on the spectra and the corresponding fragmentation patterns are indicated on the molecules themselves. (For interpretation of the references to color in this figure legend, the reader is referred to the web version of this article.)

Studies of the formation of turnover products from CYP152K6-dependent oxidation of dodecanoic acid, tetradecanoic acid and hexadecanoic acid were done as described in the Methods section. No products were formed using hexadecanoic acid as substrate. In the case of tetradecanoic acid, only a small amount of the 2-hydroxylated form could be detected, with large amounts of substrate remaining. However, using dodecanoic acid as substrate we observed the formation of predominantly 2-hydroxydodecanoic acid (77% of total product), and only trace amounts of 3-hydroxydodecanoic acid. There is no evidence of the formation of a decarboxylated alkene product (i.e. 1-undecene). Secondary products are also observed, namely undec-1-en-1-ol (3.5%), 2-hydroxydodec-2-enoic acid (1.5%), and 2,3-dihydroxydodecanoic acid (5%), with ~13% unconverted dodecanoic acid remaining at the end of the reaction (Fig. 5).

To establish whether the 2,3-dihydroxydodecanoic acid secondary product results from a second hydroxylation of 2-hydroxydodecanoic acid or of 3-hydroxydodecanoic acid (or from both of these substrates), further turnover reactions were done using the two different mono-hydroxylated products. These studies revealed that 2-hydroxydodecanoic acid could be converted to the 2,3-dihydroxydodecanoic acid by further oxidation with CYP152K6, while no 2,3-dihydroxydodecanoic acid (or other products) were formed using 3-hydroxydodecanoic acid as substrate. Thus, only 2-hydroxydodecanoic acid is a viable substrate for the formation of 2,3-dihydroxydodecanoic acid. In addition, the formation of undec-1-en-1-ol and 2-hydroxydodec-2-enoic acid is also observed using 2-hydroxydodecanoic acid as the substrate. CYP152K6 is evidently capable of both desaturation and decarboxylation reactions. In the case of the further conversion of the 2-hydroxydodecanoic acid it is likely that a 2,3-desaturation reaction occurs first to generate the 2-hydroxydodec-2-enoic acid, followed by oxidative decarboxylation of this secondary product to produce the undec-1-en-1-ol [32]. Other studies established that undec-1-en-1-ol could not be formed from 1-undecene by CYP152K6.

### 3.5 Structural analysis of CYP152K6

**Fig. 6:**
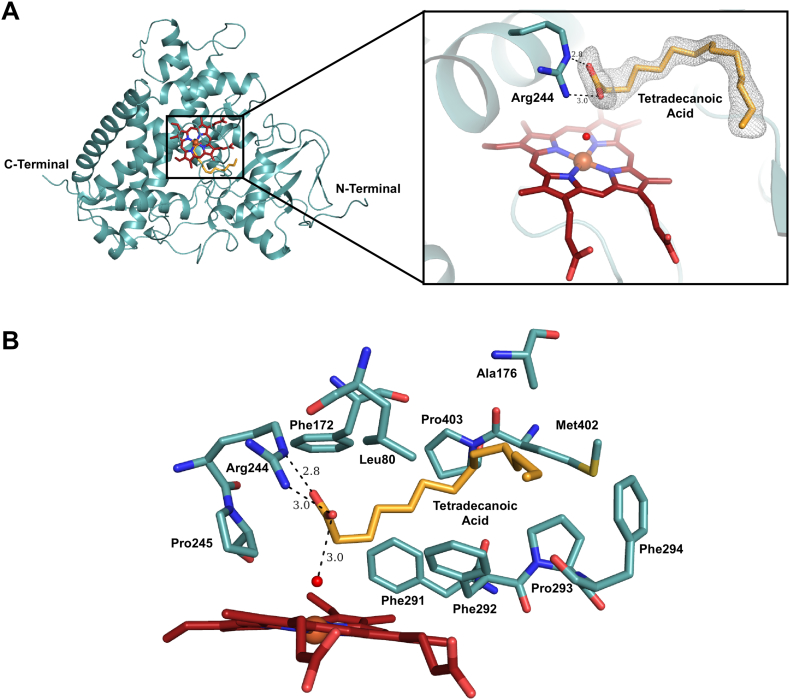
Active site structure and substrate binding mode in CYP152K6. Panel A shows a cartoon representation of the overall structure of CYP152K6 from *B. methanolicus*. The inset shows the bound tetradecanoic acid substrate in orange sticks while the heme prosthetic group is depicted in red sticks. Dashed lines show the hydrogen-bonding interactions and the distances in Angstroms (Å). The simulated annealing mFo-DFc omit map (contoured at 3.0 σ) is shown for the bound tetradecanoic acid as a gray mesh. The axial water ligand is shown as a red sphere. Panel B shows a close-up view illustrating the hydrophobic interactions made between tetradecanoic acid and neighbouring residues in the active site pocket. Interactions are also made between the substrate carboxylate group and Arg244 and the distal water molecule.

**Fig. 7:**
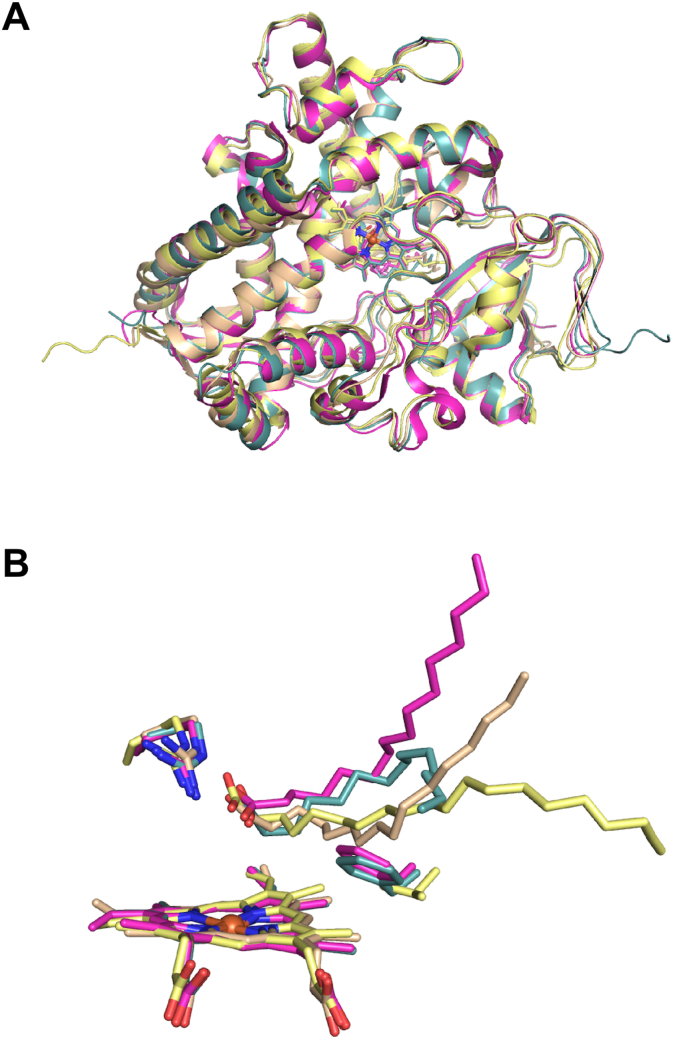
Structural comparisons of different CYP152 family members. Panel A shows the superimposed structures of (i) CYP152K6 (blue, PDB: 6FYJ), (ii) P450_BSβ_ (CYP152A1, wheat, PDB: 1IZO), (iii) P450_SPα_ (CYP152B1, magenta, PDB: 3AWM) and (iv) OleT_JE_ (CYP152L1, yellow, PDB: 4L40). Panel B shows superimposed structures of the same P450 enzymes showing the bound fatty acid substrates in the active-site: (i) tetradecanoic acid (C14:0), (ii) palmitoleic acid (C16:1) (iii) hexadecanoic acid (C16:0) and (iv) eicosanoic acid (C20:0). The conserved arginine residue and Phe292, and their equivalent residues in other peroxygenases, are also shown as sticks.

The crystal structure of CYP152K6 was solved to a resolution of 1.3 Å with good stereochemical quality. Data collection and refinement statistics are shown in Table 1. The protein crystallized with a single monomer in the asymmetric unit and showed good electron density for the entire protein molecule. The overall protein fold and structure closely resembles other members of the CYP152 family of P450 peroxygenases (Fig. 6A). The structure of the CYP152K6 P450 superimposes well with those of other CYP152 peroxygenases, and with an average root mean square deviation of only 0.85 Å for all Cα backbone atoms (Fig. 7A). Inspection of the active site revealed additional density, consistent with the binding of a fatty acid substrate above the plane of the heme prosthetic group. Indeed, addition of tetradecanoic acid (C14:0) to the model and subsequent refinements confirmed the presence of a bound fatty acid substrate in the active site (Fig. 6A). The bound tetradecanoic acid (as discussed above) does not cause any substantial change in the UV–visible spectrum of CYP152K6 (as is also the case for fatty acid binding to the P450_SPα_ enzyme) [30]. As a consequence, the binding of tetradecanoic acid became evident only following the determination of the CYP152K6 crystal structure. Tetradecanoic acid binding to CYP152K6 must occur during the production of the P450 in the *E. coli* expression cells, and its affinity is sufficiently strong that CYP152K6 crystallizes with tetradecanoic acid bound. No fatty acids were introduced at any point during the protein purification regime, and the crystallization conditions also did not contain any lipids. A similar observation was made recently for the CYP152N1 peroxygenase P450 from *Exiguobacterium* sp. AT1b [34]. The binding of tetradecanoic acid to CYP152K6 is evidently very strong, since attempts to remove the lipid either by extensive dialysis or by passing the CYP152K6 sample through a Lipidex column had little effect. Addition of hexadecanoic acid with H_2_O_2_ did not result in any significant product formation, while small amounts of 2-hydroxytetradecanoic acid were formed from tetradecanoic acid-bound CYP152K6. However, dodecanoic acid was able to displace tetradecanoic acid and a number of oxidized species were obtained from primary and secondary turnovers of this substrate.

**Table 1:** Data collection and refinement statistics.

	CYP152K6 Tetradecanoic acid bound
PDB ID	6FYJ
Data collection	
Space group	*C*2
Cell dimensions	
*a*, *b*, *c* (Å)	93.9, 71.7, 68.4
α, β, γ (°)	90.0, 103.6, 90.0
Resolution range (Å)	56.14–1.3
*R*_meas_	0.043 (1.5)^a^
*I*/σ*I*	14.4 (1.0)
Completeness (%)	99.0 (95.3)
Redundancy	2.9 (2.0)
Refinement	
Resolution (Å)	56.14–1.30
No. reflections	105732
*R*_work_/*R*_free_ (%)	17.3/19.3
No. of residues	
Protein	417
Water	440
Ligand/ion	1 heme, 1 tetradecanoic acid, 1 PEG
*B*-factors (Å^2^)	
Protein	21.1
Ligand	20.1
R.m.s. deviations	
Bond lengths (Å)	0.014
Bond angles (°)	1.336
Ramachandran	
Favoured (%)	97.8
Outlier (%)	0.5

a Values in parentheses are for highest-resolution shell.

An arginine residue, Arg244 in CYP152K6, is phylogenetically conserved in the CYP152 family of P450 peroxygenases, and makes strong hydrogen-bonding interactions with the carboxylate group of tetradecanoic acid. There are also extensive interactions made by several hydrophobic residues lining the active-site pocket that further stabilize the alkyl chain of tetradecanoic acid (Fig. 6B). The bound ligand is positioned in the active site in a binding mode that is above the plane of the heme, and with the Cα and Cβ carbons of tetradecanoic acid in close proximity of the heme iron (at distances of 4.8 Å and 5.5 Å, respectively). The distortion of the heme A and B pyrrole groups of the CYP152K6 heme prosthetic group is clearly visible, and this type of structural perturbation is highly similar to that observed for the OleT_JE_ P450 heme group. This distortion of the heme group is believed to move these pyrrole rings closer to the substrate [35]. However, in absence of a substrate-free CYP152K6 structure it is unclear whether this heme distortion is dependent on substrate binding or not. A water molecule at a distance of ~2.1 Å directly above the heme iron acts as the 6th (distal) ligand, with a cysteine thiolate providing the 5th (proximal) ligand. This results in the heme having a hexacoordinated state in the substrate-bound CYP152K6 structure. This is also consistent with observations from UV–visible spectroscopy, where the purified enzyme is clearly in a low-spin, ferric hexacoordinated state. Interestingly, the distal water molecule is also within hydrogen-bonding distance (~3.0 Å) of one of the oxygen atoms from the carboxylate group of tetradecanoic acid.

The active site architecture of CYP152K6 is very similar to those of other peroxygenases in the CYP152 family. As seen in the substrate-bound structures of P450_BSβ_ and P450_SPα_, the fatty acid substrate-binding pocket of CYP152K6 has a narrow access channel to the solvent, which is flanked by the FG-loop and the C-terminal loop regions. Structural comparisons of the tetradecanoic acid-bound CYP152K6 enzyme with other CYP152 family enzymes highlight considerable differences in the binding modes of the fatty acid substrates in the active site (Fig. 7B). For example, in both P450_BSβ_ and P450_SPα_ the fatty acids occupy the elongated substrate-binding pocket with their respective alkyl fatty acid tails protruding out towards the narrow solvent access channel. In contrast, the CYP152K6 tetradecanoic acid alkyl chain has a non-linear configuration in the active site. The alkyl chain bends by ~90° around the C8 atom of tetradecanoic acid to produce a well-defined structural “kink” in the lipid chain. This spatial placement of the substrate in the active site might be of significance, as it has been proposed that positioning of the substrates in the active site plays a critical role in determining the chemistry of the catalytic reaction, i.e. whether hydroxylation or decarboxylation of the fatty acid will take place [36].

Furthermore, in P450_SPα_ Phe288 has been found to play a critical role in ensuring hydroxylation at the α position of the fatty acid substrate [29,37]. The equivalent residue in CYP152K6 is also a phenylalanine, Phe292, which is involved in making hydrophobic interactions with C3 (Cβ), C5 (Cδ) and C13 carbon atoms of the bound tetradecanoic acid molecule. Phe292 occupies the space between the heme and the tetradecanoic acid, and the kink in the alkyl chain of tetradecanoic acid appears around this residue. The corresponding residues in this position in P450_BSβ_ and OleT_JE_ are glycine and valine, respectively. Recently, another peroxygenase from the CYP152 family, CYP152N1 from *Exiguobacterium* sp. ATIb, has been characterized and was found to be an α-(*S*)-selective fatty acid hydroxylase [34]. This enzyme also has a phenylalanine at the equivalent Phe292 position. The crystal structure of CYP152N1 also revealed a bound molecule of tetradecanoic acid in the active site, and this lipid also exhibited a similar distortion in its alkyl chain to that observed in CYP152K6.

## 4 Discussion

CYP152K6 from *Bacillus methanolicus* MGA3 is an interesting addition to the growing family of CYP152 peroxygenases. These enzymes typically share ~30–50% amino acid identity and have largely conserved structures. Minor differences in the active site structure and in the binding orientation of the fatty acid substrate dictate the chemistry of the peroxygenase reaction and the relative amounts of the primary products (alpha- and beta-hydroxylated fatty acids and terminal alkenes). In the case of CYP152K6 the predominant product formed in turnover of dodecanoic acid was the 2-hydroxydodecanoic acid at ~77% of the overall product, with only a small proportion of 3-hydroxydodecanoic acid and no detectable terminal alkene product formed (Fig. 5). However, three other secondary products are formed by the action of CYP125K6 on the primary products, producing undec-1-en-1-ol, 2-hydroxydodec-2-enoic acid, and 2,3-dihydroxydodecanoic acid species. The P450 peroxygenases are known to catalyze desaturation reactions in addition to hydroxylation and decarboxylation, and it is likely that a 2,3-desaturation reaction converts 2-hydroxydodecanoic acid into 2-hydroxydodec-2-enoic acid, prior to a decarboxylation reaction to form undec-1-en-1-ol. Importantly, the 2,3-dihydroxydodecanoic acid secondary product is a novel compound produced by the CYP152K6 peroxygenase, and must result from consecutive oxidations at the 2- and 3-positions on dodecanoic acid. To address the issue of whether one or both of the primary products (2-hydroxydodecanoic acid and 3-hydroxydodecanoic acid) could undergo a secondary hydroxylation to form 2,3-dihydroxydodecanoic acid, further analytical studies were undertaken using both 2-hydroxydodecanoic acid and 3-hydroxydodecanoic acid as substrates. The 2,3-dihydroxydodecanoic acid product was found to accumulate only in the reaction with 2-hydroxydodecanoic acid as substrate, indicating that CYP152K6 can catalyze the 3-hydroxylation of 2-hydroxydodecanoic acid, but that 2-hydroxylation of 3-hydroxydodecanoic acid is not feasible.

It is interesting to note that while dodecanoic acid is a good substrate for CYP152K6, only very small amounts of 2-hydroxylated product are formed with tetradecanoic acid, and no detectable product was formed with hexadecanoic acid. This may imply that the enzyme favours the binding of shorter chain fatty acids. While many P450s (including some peroxygenase P450s such as OleT_JE_/CYP152L1) undergo a low- to high-spin conversion with associated heme spectral changes on the binding of fatty acid substrates, neither CYP152K6 nor P450_SPα_ show any substantial heme spectral conversion on binding with fatty acids. This presents problems in determining the binding affinity using UV–visible spectroscopy methods, and thus further studies on the CYP152K6 enzyme will explore isothermal titration calorimetry (ITC) as a route to confirming substrate binding and to determine dissociation constants (*K*_d_ values) for lipid substrates and their products (where feasible). This approach should also be useful in identifying fatty acid substrates of shorter chain length that could be tested for product formation using GC–MS or other methods.

The crystal structure of CYP152K6 was determined to high-resolution (1.3 Å) following the expression and purification of the P450 from an *E. coli* host strain. The structure clearly reveals the binding of the C14 fatty acid tetradecanoic acid, despite there being no such lipid in the crystallization conditions used, and no lipid added at any stage during the purification process. The substrate must have been incorporated into the CYP152K6 active site during the process of enzyme production in the *E. coli* cells. The tetradecanoic acid carboxylate group interacts with nitrogen atoms on a phylogenetically conserved arginine residue (Fig. 6A), as is also observed in a number of other structures of substrate-bound forms of peroxygenases, e.g. OleT_JE_ [35]. The tetradecanoic acid binding is stabilized by several interactions with hydrophobic amino acids in the active site cavity and is positioned close enough to the heme such that oxidative catalysis would be expected to occur. The fact that the CYP152K6/tetradecanoic acid complex is stabilized following purification of the enzyme may be due in part to the removal of potential *E. coli* redox partners and hydrogen peroxide. However, it should be noted that only a small amount of hydroxylated tetradecanoic acid was formed in the in vitro reaction of CYP152K6 with this substrate. This might again point to the tight binding of tetradecanoic acid to CYP152K6, as evident from its co-purification with the substrate. Potentially, the apparent weak turnover of the C14:0 and C16:0 fatty acids may be associated with their longer chain lengths and the greater number of stabilizing bonds made with CYP152K6 hydrophobic residues (as well as the key fatty acid carboxylate-arginine interaction), and could thus result from e.g. a slow product dissociation rate constant (*k*_off_).

**Fig. 8:**
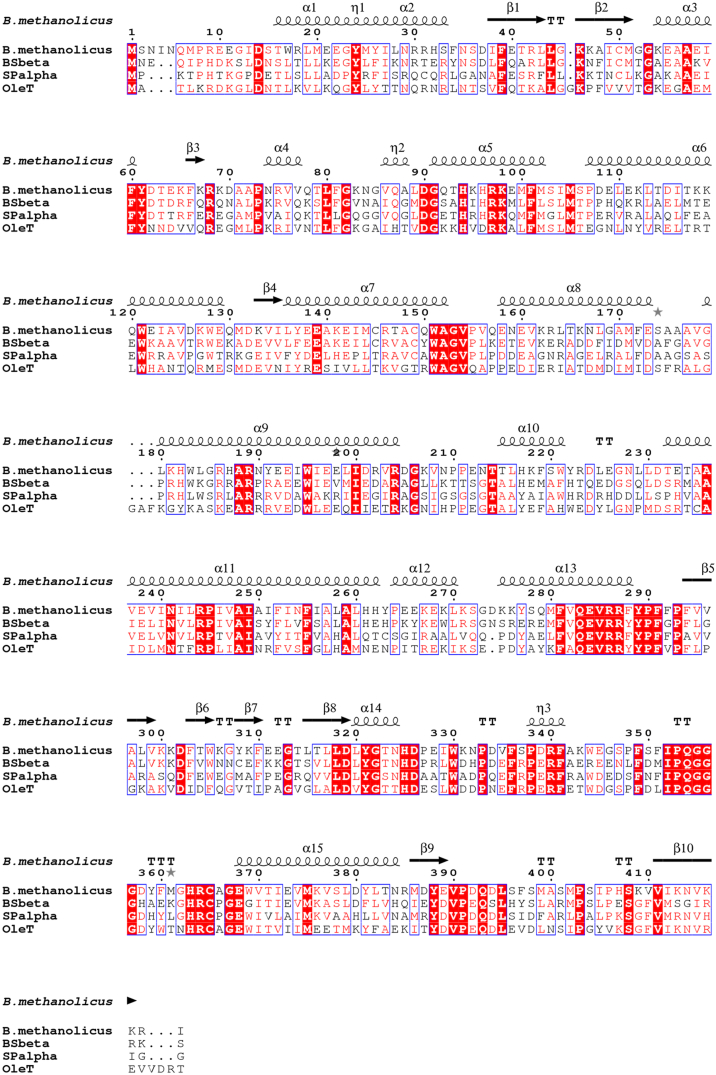
Structure based sequence alignment of different CYP152 peroxygenase family members. Strongly conserved regions are highlighted in red and the secondary structure of CYP152K6 is shown above the alignment.

Fig. 8 shows an amino acid alignment of CYP152K6 with P450_SPα_, P450_BSβ_ and OleT_JE_. There are clearly strong structural relationships between all of these peroxygenases, and a notable pair of mutations occurs at residues 244/245 – where the acid/alcohol pair found in typical monooxygenase P450s (e.g. Asp251/Thr252 in the camphor hydroxylase P450cam, CYP101A1) that is involved in proton delivery to heme iron-oxo species in the catalytic cycle is replaced by Arg244/Pro245 in each of the aligned peroxygenases. Arginine 244 is the residue involved in binding the fatty acid carboxylate group, and hence these mutations reinforce the apparent evolutionary conversion of these enzymes from monooxygenases to peroxygenases.

In conclusion, we present structural, catalytic and spectroscopic data on the *Bacillus methanolicus* CYP152K6, a new member of the CYP152 family of peroxygenase P450s. The high-resolution CYP152K6 crystal structure provides insights into the binding mode of the substrate tetradecanoic acid (Fig. 6, Fig. 7). However, tetradecanoic acid appears to be a poor substrate that is barely turned over by CYP152K6. In contrast, dodecanoic is a much better substrate and produces five different products, three of which arise from secondary turnover of the primary product 2-hydroxydodecanoic acid. A novel peroxygenase product, 2,3-dihydroxydodecanoic acid, is formed exclusively by the 3-hydroxylation of 2-hydroxydodecanoic acid. Future work will focus on studies of CYP152K6's ability to oxidize fatty acids shorter than C12, and in view of the much more effective CYP152K6-dependent oxidation of dodecanoic acid compared to the longer chain fatty acids tested.

## Abbreviations

BSTFA: *N*,*O*-Bis(trimethylsilyl)trifluoroacetamide
CPR: cytochrome P450 reductase
EPR: electron paramagnetic resonance spectroscopy
IPTG: isopropyl-β-d-thiogalactopyranoside
P450: cytochrome P450
TCMS: trimethylchlorosilane
